# Managerial preventions of common mental disorders and the association with stigmatizing attitudes

**DOI:** 10.1093/eurpub/ckac131.262

**Published:** 2022-10-25

**Authors:** J Hultqvist, P Zhang, C Staland-Nyman, M Bertilsson

**Affiliations:** 1 Department of Health and Rehabilitation, University of Gothenburg, Göteborg, Sweden; 2 School of Public Health and Community Medicine, University of Gothenburg, Göteborg, Sweden; 3 School of Health and Welfare, Halmstad University, Halmstad, Sweden

## Abstract

**Background:**

Common mental disorders (CMDs) are an extensive problem in the society and attributed to stigma. Prevention of CMD at work is advocated but few studies have investigated what kind of preventive actions managers take. We investigated managers’ attitudes to depression and two managerial preventive actions (MPA): ‘reviewing assignments and the work situation’ (MPA-review) and ‘taking initiative to talk about depression and anxiety at the workplace’ (MPA-talk). We hypothesized that managers’ negative attitudes towards depression would be negatively associated with both MPAs.

**Methods:**

An on-line survey was sent in 2017 to 4737 managers, answer rate 71% (n = 3358), of which 2 899 were included in this study. Negative attitudes were measured through the 12-item instrument “Managerial stigma towards employee depression” (scores 12-72), a cut-off at the 3rd quartile was used as an indicator for having negative attitudes. MPAs were measured with two single questions. Negative attitudes to depression were analyzed in relation to MPA-review and MPA-talk using binary logistic regression analysis with adjustments for sex, education, managerial experience and training, lived experiences of CMD, work organizational context and general preventive actions in the organization towards CMD.

**Results:**

The proportion of managers with negative attitudes to depression was 20%, performing MPA-review and MPA-talk was 50% and 57% respectively. Adjusted for all co-variates, managers with negative attitudes towards employees with depression were less likely to do both MPA-review (OR 0.71; 95% CI, 0.57-0.89) and MPA-talk (OR 0.53; 95% CI, 0.42-0.66).

**Conclusions:**

Managers with negative attitudes to depression were less likely to take actions to prevent CMD among their employees which confirmed the study hypotheses. The study suggests that initiatives to reduce stigma among managers could be a way forward to prevent CMD at work.

**Key messages:**

• Stigma to depression hampers managers’ prevention of CMD and needs to be addressed.

• To increase managers prevention of CMD, managerial training to reduce stigma towards depression is essential.

